# Single-cell transcriptomics in cancer: computational challenges and opportunities

**DOI:** 10.1038/s12276-020-0422-0

**Published:** 2020-09-15

**Authors:** Jean Fan, Kamil Slowikowski, Fan Zhang

**Affiliations:** 1 Department of Biomedical Engineering, Johns Hopkins University, Baltimore, Maryland USA; 2 Center for Immunology and Inflammatory Diseases, Massachusetts General Hospital, Charlestown, MA USA; 3 Center for Data Sciences, Brigham and Women’s Hospital, Boston, MA USA; 4 Department of Biomedical Informatics, Harvard Medical School, Boston, MA USA

**Keywords:** Cancer, Computational biology and bioinformatics

## Abstract

Intratumor heterogeneity is a common characteristic across diverse cancer types and presents challenges to current standards of treatment. Advancements in high-throughput sequencing and imaging technologies provide opportunities to identify and characterize these aspects of heterogeneity. Notably, transcriptomic profiling at a single-cell resolution enables quantitative measurements of the molecular activity that underlies the phenotypic diversity of cells within a tumor. Such high-dimensional data require computational analysis to extract relevant biological insights about the cell types and states that drive cancer development, pathogenesis, and clinical outcomes. In this review, we highlight emerging themes in the computational analysis of single-cell transcriptomics data and their applications to cancer research. We focus on downstream analytical challenges relevant to cancer research, including how to computationally perform unified analysis across many patients and disease states, distinguish neoplastic from nonneoplastic cells, infer communication with the tumor microenvironment, and delineate tumoral and microenvironmental evolution with trajectory and RNA velocity analysis. We include discussions of challenges and opportunities for future computational methodological advancements necessary to realize the translational potential of single-cell transcriptomic profiling in cancer.

## Cancer: analyzing the RNA of single cells

By analyzing gene expression patterns in individual tumor cells, researchers can gain patient-specific insights that might inform more effective cancer treatment. Tumors are highly dynamic and heterogeneous collections of cells. Single-cell transcriptomics techniques can offer a valuable window into that complexity but only if the appropriate computational tools are used to analyze the data. Jean Fan of Harvard University, Cambridge, USA, and colleagues have reviewed some of these computational strategies and how they can be employed in cancer research. Single-cell analysis algorithms, for example, can reveal characteristics that distinguish healthy cells from cancerous cells, or indicate how the cells within the tumor may be communicating with each other to promote malignant growth. These are still new technologies, however, and the authors highlight the limitations of the conclusions that can currently be drawn from such analyses.

## Introduction

Cancer is a highly heterogeneous disease exhibiting phenotypic diversity driven by molecular aberrations at the genetic, epigenetic, transcriptomic, and protein levels in cells that interact within distinctly spatially organized microenvironments^1^. Such heterogeneity presents challenges to current standards of treatment by contributing to metastasis and therapeutic resistance, which ultimately impact clinical outcomes. Accurate characterization of this heterogeneity is essential for delineating the mechanisms of cancer pathogenesis, developing effective treatment strategies, and identifying novel targets for immunotherapy and drug development^2^.

The characterization of heterogeneity at the transcriptomic level has been promising, as changes in transcriptional activity and regulation generally underlie cellular phenotypic diversity. The continuous advances in next-generation sequencing technologies such as RNA sequencing (RNA-seq) have enabled the genome-wide quantification of gene-expression levels in a high-throughput manner under diverse conditions. Over the years, these data have led to numerous discoveries in biology, including insights into the phenotypic consequences of molecular aberrations in cancer^3^.

**Fig. 1:**
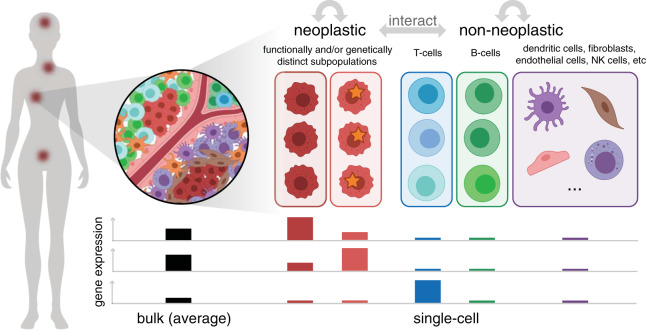
Cancer may manifest as multiple spatially distinct tumors composed of multiple functionally and/or genetically distinct neoplastic subpopulations as well as diverse nonneoplastic cell types and states that interact to impact clinical outcomes. In this illustration, (left) a single patient presents with cancer at multiple sites. (top right) Zooming into one site, multiple cell types, both neoplastic and nonneoplastic, can be observed. Within the neoplastic cells, two distinct subpopulations marked by different somatic mutations, indicated with a star, are present. Likewise, within nonneoplastic cells, multiple distinct T-cell and B-cell subtypes and states, indicated with different shades of colors, are present. (bottom) Bulk measurements provide the average quantification of gene expression, which potentially obscures proportional and subpopulation or state-specific differences. The expression levels of three genes determined from bulk and pooled single-cell measurements for the two different neoplastic subtypes and major nonneoplastic cell types (T-cells, B-cells, and other) are illustrated as an example. The first two genes appear to be highly expressed in the bulk sample but are actually highly expressed in only one of the neoplastic subpopulations. In contrast, the third gene appears to show low expression in the bulk sample but is actually highly expressed among T-cells, which are proportionally low in abundance. In this manner, single-cell measurements could enable the finer, unbiased characterization of transcriptional features that underlie important cancer phenotypes.

However, such transcriptomic profiling studies have conventionally involved bulk RNA-seq analysis of pooled, heterogeneous mixtures of cells from cancer samples (Fig. 1). Thus, the resulting gene expression quantification results represent average values across large mixtures of cells and are influenced by the particular transcriptional profiles as well as the abundance of different cell types and states within that sample. Even for samples of sorted cell subsets, finer aspects of heterogeneity, such as transcriptional differences between distinct subpopulations, can still be missed if these subpopulations are in the same sorted subset. In contrast, transcriptomic profiling at a single-cell resolution in cancer offers the opportunity to identify and characterize transcriptionally distinct subpopulations and states that may impact clinical outcomes, inform treatment strategies, or point to new therapeutic opportunities^4,5^.

**Fig. 2:**
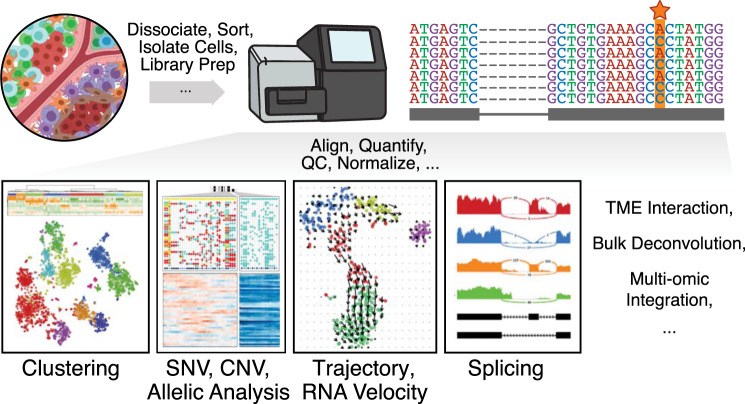
Single cell RNA-seq workflow and downstream computational analyses. High-throughput single-cell transcriptomic technologies such as single-cell RNA sequencing generally begin with experimental workflows tailored to distinct tumor and tissue types (dissociating, sorting, and isolating cells, etc.), which ultimately result in sequences that can be aligned, quantified, quality control (QC) filtered, and normalized in different ways to enable a number of downstream computational analyses, such as clustering analysis to identify transcriptionally distinct cell types and subpopulations, allelic analysis to identify single nucleotide variants (SNVs, indicated with a star in the read pileup) or copy number variants (CNVs), trajectory analysis, splicing detection, or the inference of tumor-microenvironmental (TME) interactions.

To enable transcriptomic profiling at a single-cell resolution, a number of high-throughput single-cell RNA-sequencing (scRNA-seq) protocols, platforms, and technologies have been developed^6–10^ and reviewed^11–14^. In terms of computational processing, each particular scRNA-seq protocol, platform, and technology may demand different read processing, quality control, and normalization procedures^15^. Despite these differences, there are a number of common downstream computational analyses that can be applied (Fig. 2). Here, we highlight computational methods for performing a number of analyses relevant to cancer research, including (1) identifying common cell types and states shared across patients and disease states from multiple scRNA-seq datasets; (2) distinguishing neoplastic from nonneoplastic cells using marker and fusion gene detection, copy-number variation inference, and somatic mutation calling from scRNA-seq data; (3) inferring cell–cell communication from the expression of genes encoding receptors and ligands; (4) estimating the proportions of cell types in bulk gene expression profiles; and (5) characterizing transcriptional dynamics using trajectory inference and RNA velocity analysis.

### Unified analysis across many patients and disease states

**Fig. 3:**
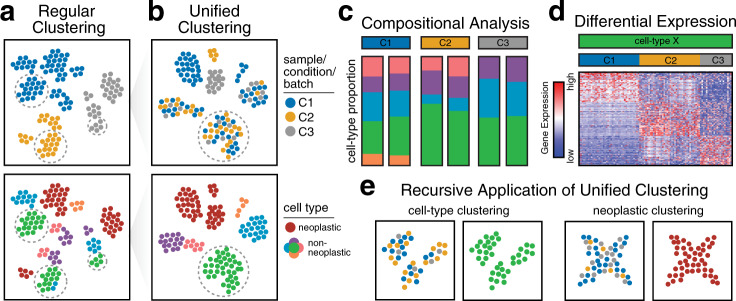
Unified clustering analysis. **a** The clustering of cells from different samples across diverse conditions may result in cells being aggregated by sample, condition, or other technical factors such as the batch rather than the cell types of interest. The top illustration shows a 2D reduced dimensional representation (e.g., tSNE) in which each point is a cell and is colored according to the sample, condition, or batch label. The bottom illustration shows the same 2D embedding colored according to cell type. Cells are aggregated according to the sample, condition, or batch, rather than the cell type, making the identification of shared cell types difficult. **b** Unified clustering analysis results in cells that are appropriately aggregated by cell type, particularly for nonneoplastic cell types. **c** After the identification of common cell types, additional downstream analyses may be performed. For example, compositional analysis comparing nonneoplastic cell-type proportions across three conditions, each with two replicates, can be performed to show high correspondence within replicates but differences across conditions. **d** Differential expression analysis can also be applied to one cell type, comparing each condition to all others, identifying differentially upregulated genes in each condition. **e** Unified clustering analysis may be applied recursively to identify additional subtypes or states within nonneoplastic cell types (left) or shared transcriptional states among neoplastic cells across patients.

In the context of cancer, the analysis of single-cell transcriptomics data is often complicated by elaborate study designs that may include samples from individuals with and without disease, multiple samples from the same individual collected at different time points (e.g., pretreatment and posttreatment), or multiple samples from different individuals exhibiting diverse disease states. Such study designs can enable the discovery of transcriptional characteristics shared across patients that may define commonly perturbed molecular pathways in disease. However, the identification of shared cell types or states in data from complex study designs may be difficult when cells are clustered by sample or batch instead of the cell types or states of interest^16^ (Fig. 3a). This challenge regarding batch effects can lead to false discoveries^17^ and complicates the identification of shared cell types and states that are necessary for downstream analyses and biological interpretation. Analyses dependent on the identification of shared cell types and states are further complicated by the presence of truly unique patient-specific differences inherent to cancer. Although care should be taken to plan experimental designs that minimize batch effects—for example, some recent studies have used multiplexed scRNA-seq to pool cells from multiple samples into a single batch for sequencing^18–21^—this may not be possible in practice due to logistical limitations concerning sample acquisition, time constraints, and limitations of sample processing and handling.

To facilitate the identification of shared cell types and states across datasets, a number of well-established batch correction methods have been developed for bulk RNA-seq data to remove batch effects by adjusting gene-expression levels to make data from different batches more comparable^22,23^. Batch correction is more difficult for scRNA-seq data in which each sample may contain different abundances of cell types, each with distinct gene expression profiles. Therefore, it may not be appropriate to apply the same global adjustment to all cells in a sample. As an alternative approach, many computational methods have been developed for the unified analysis of multiple scRNA-seq datasets that explicitly model or implicitly control for sample and batch-specific differences to identify shared aspects of transcriptional variation across datasets.

Computational methods for unified single-cell transcriptomics analysis generally search for shared aspects of transcriptional variation that can be aligned across datasets from multiple samples, batches, or conditions (Fig. 3b). Despite differences in their implementation and algorithms, many methods share a similar conceptual framework: each method begins by reducing the dimensionality^24^ of the normalized gene-expression data to a smaller set of features (e.g., latent space), aligning these features across datasets, using the aligned features to identify clusters of cells (that may be interpreted as cell types), and finally using the aligned features and identified clusters as the input for 2D visualization algorithms. For example, MultiCCA^25^ identifies shared aspects of variation between pairs of datasets by iteratively applying canonical correlation analysis^24^ to two datasets at a time and adding additional samples at each iteration. The canonical components are then adjusted using dynamic time warping and serve as the input for graph-based clustering algorithms and 2D visualization. Mutual nearest-neighbor (MNN) Correct^26^, Scanorama^27^, and Conos^28^ build MNN graphs between cells from different datasets, where two cells are connected in the graph if they are transcriptionally similar. These MNN graphs can then be used directly to derive unified cluster annotation, in the case of Conos, or applied to adjust the data prior to serving as the input for clustering algorithms, in the case of MNN Correct and Scanorama. A major limitation of MultiCCA and MNN Correct is that these methods can produce different results depending on the ordering of the datasets in the analysis^27^. To overcome the ordering limitation, Scanorama automatically finds a favorable order, while Conos builds a joint MNN graph of all datasets. However, when sample and data acquisition occur continuously and in parallel with the analysis, approaches such as MultiCCA, MNN Correct, and Conos may make it more computationally efficient to incorporate additional datasets into the unified analysis without rerunning all analyses on previously analyzed datasets. In contrast, LIGER^29^ uses integrative nonnegative matrix factorization^30^ (NMF) to split the full expression matrix (of all datasets) into two parts: a matrix of shared factors and a matrix of dataset-specific (batch-effect) factors. Then, the shared factors serve as the input for graph-based clustering algorithms and 2D visualization. A notable limitation of all the aforementioned methods is the use of a single categorical variable to encode batch labels. The accommodation of multiple variables could be relevant for cancer data analyses to enable the identification of shared cell types and states across datasets with additional clinically relevant features (e.g., patient sex, age, and genetics) or additional dimensions such as time points (e.g., pretreatment and posttreatment, time series) or drug dosages. To this end, Harmony^31^ is able to accommodate multiple categorical variables to encode batch information. Harmony^31^ iteratively identifies clusters of cells and applies local linear adjustments to these cells while maximizing the diversity of batches within clusters. Such unified analyses with Harmony have been applied to scRNA-seq datasets of hepatocellular carcinoma tumors and immune-relevant sites from multiple patients and platforms to identify T-Regs, exhausted CD8+ T cells, and subtypes of macrophages and DCs that are shared across patients and enriched in cancer samples^32^.

Alternatively, rather than explicitly taking into consideration batch information, other computational methods for unified single-cell transcriptomics analysis learn a function that maps a dataset onto a low-dimensional latent space and then apply this function to map datasets from different samples or batches onto the same space. For example, scCoGAPS^33^ uses Bayesian NMF with prior distributions designed to handle scRNA-seq data to discover latent spaces in a reference scRNA-seq dataset and then uses projectR^33^ to project new scRNA-seq data onto the learned latent spaces. Such methods thus rely on identified latent spaces being free of batch effects rather than explicitly controlling for batch-specific differences. Such methods may be particularly computationally efficient in the construction of a large reference or atlas model that can then be applied to cells from a smaller dataset under the assumption that the initial reference model contains all possible cell types and states.

While the reliability of many unified single-cell transcriptomic analysis methods has been tested by integrating datasets from many non-diseased tissues^34^, their application to datasets from patients with cancer raises additional concerns. The aforementioned computational methods for the unified analysis of scRNA-seq datasets work best when all datasets contain common cell types or states in similar proportions. For example, MultiCCA and MNN Correct assume that all datasets contain at least one shared cell type. However, due to prevalent inter- and intratumoral heterogeneity, this assumption may no longer be valid in a cancer setting. As such, these methods may result in overcorrection when different cell types from different samples are assigned to the same cluster in the unified analysis and misinterpreted as the same cell type. In addition to discrete cell types and cell states, cancer datasets may also contain cells exhibiting smooth developmental and evolutionary trajectories. Unified analysis methods may result in another form of overcorrection when dataset integration fails to preserve the topology of these biological trajectories^31^. Analyzing each dataset individually using cluster annotations from unified analysis can help to assess the quality of the unified results.

After the identification of common cell types and states, additional compositional comparisons or differential expression analyses can be applied to characterize the changes between different treatments, disease stages, or other conditions. For example, generalized linear models have been used to identify differential abundances in cell-type proportions by comparing cases versus controls^35,36^ (Fig. 3c) and to identify differentially expressed genes across culture conditions^37^ (Fig. 3d) while accounting for important covariates using fixed effects for variables such as sex and age and random effects for the patient and batch. Accounting for covariates in a linear model with unadjusted gene-expression data should be preferred for differential expression analysis with adjusted gene-expression data to avoid the identification of spuriously significantly differentially expressed genes^38^.

Alternatively, newer approaches for unified single-cell transcriptomics analysis based on deep neural networks have been developed to enable batch correction, normalization, imputation, dimensionality reduction, and clustering for millions of cells simultaneously by fitting a single generative model^39–41^. For example, scVI^39^ uses deep neural networks to learn the parameters of a hierarchical Bayesian model that is designed to separate biological signals from unwanted factors (e.g., batch effects) while embedding the cells in a low-dimensional latent space. The resulting latent space vectors can then serve as the input for clustering algorithms and 2D visualization. In contrast, SAUCIE^40^ uses a deep neural network in which some of the layers are designed to perform cluster annotation and 2D visualization, thereby eliminating the need for additional clustering analysis of the latent space vectors. Graphical processing units can also be used to fit these deep neural network models more efficiently. While these methods currently use a single categorical variable to encode batch information, they could in theory be extended to allow the inclusion of multiple categorical or continuous variables. However, in contrast to matrix factorization methods such as PCA, CCA, and NMF, in which we can examine the contributions of each gene for each factor, the latent spaces obtained from deep learning methods may not be as easily interpretable. This raises concerns such as overfitting to technical features or other unwanted aspects of variation in the data. Therefore, additional efforts are needed to demonstrate that latent spaces from deep neural networks reflect biologically and clinically relevant patterns in different cancer tissues^42^.

Once major cell types are identified across datasets, recursive clustering may be applied to identify finer cell states (Fig. 3e). Recursive clustering has been applied to stromal^43^ as well as tumor-infiltrating myeloid cells^44^ in lung cancer to first distinguish different major cell types and subsequently reanalyze each cell type independently to identify finer subtypes and states, including those that are enriched or uniquely present in cancer samples. Such recursive clustering may become more important as the number of cells in new datasets increases. In integrative analyses of cancer datasets from multiple patients, non-neoplastic cells may cluster by cell type, while neoplastic cells segregate by patient^4,45^ due to the degree of inter-patient heterogeneity for neoplastic versus nonneoplastic cells. Therefore, when performing such unified analyses across patients, neoplastic cells may need to be considered separately from nonneoplastic cells to identify shared aspects of transcriptional heterogeneity and common cell states. To guard against overcorrection, each sample should be analyzed individually to ensure that the transcriptional programs associated with the states identified from a unified analysis are also present within individual samples. Such integrative analyses using NMF^24^ have been applied to identify the gene modules that correspond to cell cycle and aberrant developmental programs that mark distinct neoplastic subpopulations and are shared across patients in studies of both diffuse midline gliomas^46^ and head and neck squamous cell carcinomas^47^.

### Distinguishing neoplastic from nonneoplastic cells

In the context of cancer, one unique analytical challenge is distinguishing neoplastic cells (e.g., tumor cells) from nonneoplastic cells (e.g., immune cells, endothelial cells, and fibroblasts). In some studies, this challenge is circumvented by enriching neoplastic cells and/or depleting nonneoplastic cells by sorting. However, sorting is sometimes impossible due to technical limitations (e.g., a lack of suitable markers). Furthermore, sorting may be undesirable when the aim is to characterize neoplastic cells in conjunction with nonneoplastic cells in the surrounding tumor microenvironment. Thus, a number of computational methods and approaches have been developed to distinguish neoplastic cells from nonneoplastic cells.

As neoplastic cells generally exhibit extensive alterations in a variety of biochemical pathways and oncogenic programs emblematic of cancer^3^, they may be sufficiently transcriptionally distinct from nonneoplastic cells that they can be segregated through clustering analysis^12^. To identify transcriptionally distinct cell clusters, a number of computational methods for analyzing individual datasets have been developed^48–52^ and reviewed^15,53,54^. Likewise, a number of computational methods for unified analysis across many datasets were described in the previous section. In the context of cancer, these cell clusters may represent different neoplastic or nonneoplastic cell types and states. While such methods can broadly identify cell clusters, annotating these clusters as either neoplastic or nonneoplastic often proves more challenging.

**Fig. 4:**
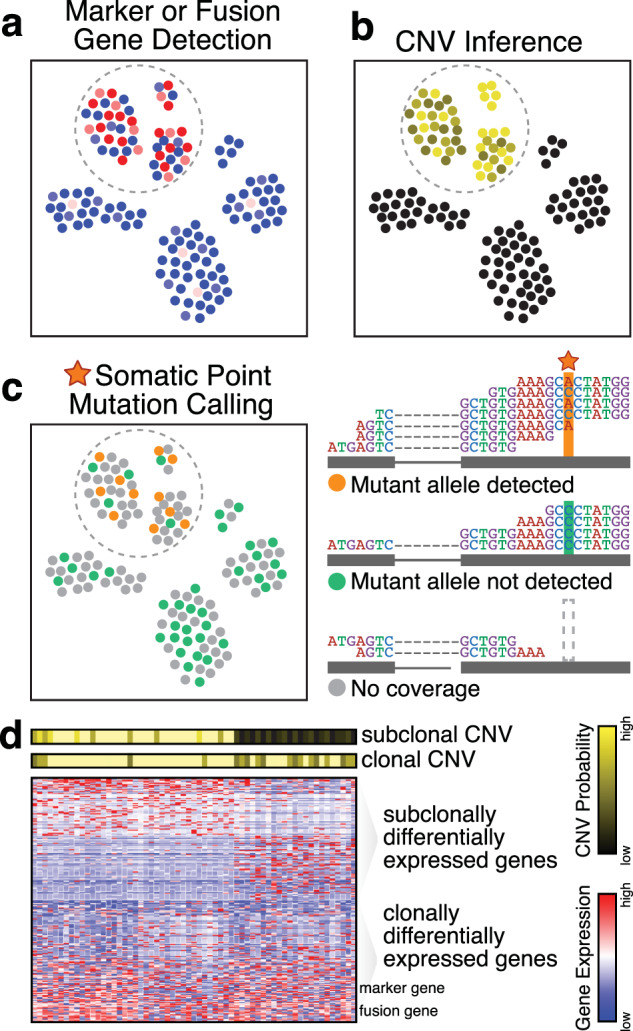
Distinguishing neoplastic and nonneoplastic cells. **a** The detection of marker or fusion genes that are uniquely upregulated or expressed in neoplastic cells may be used to identify neoplastic cells. In this illustration, many neoplastic cells exhibit high expression (red) of a marker or fusion gene, although dropouts or other technical factors result in the detection of low or no expression (blue) in other neoplastic cells in the same cluster. **b** Copy-number variant (CNV) inference may also be used to identify neoplastic cells. Normalized smoothed gene expression magnitudes and variant allele frequencies can be used to infer the probability that a cell harbors CNVs. Neoplastic cells exhibit higher probabilities of harboring any CNVs, as expected. **c** Somatic point mutation calling may be used to identify neoplastic cells. The top read pileup for a cell shows an example in which both the mutant and reference alleles are detected, indicating that the cells harbors the mutation. The middle read pileup for a cell shows an example in which the mutant allele is not detected, which could indicate that the cell does not harbor the mutation or that there is allelic dropout of the mutant allele. Alternatively, the bottom read pileup shows an example where the mutation site presents no read coverage, and thus, no mutation call can be made. **d** The inference of CNVs and other genetic alterations directly from RNA-sequencing data enables the direct interrogation of transcriptional differences among genetic subclones. A clonal CNV distinguishes neoplastic from nonneoplastic cells and is also marked by high expression of marker and fusion genes. In addition, a subclonal CNV is present. Differential expression analysis may be applied to directly identify differentially expressed genes between genetic subclones.

In certain cancers, the detection of distinct marker genes or combinations of marker genes can distinguish neoplastic from nonneoplastic cells (Fig. 4a). For example, as multiple myeloma cells are marked by CD38^+^/CD138^+^ antigen expression, they could be distinguished by the codetection of high *CD138* (*SDC1*) and *CD38* gene expression in scRNA-seq data. However, scRNA-seq data may be subject to numerous technical artifacts such as drop-outs, when a gene is expressed but not detected^55^, or high sparsity, rendering such binary classification based on marker detection liable to false negatives. Furthermore, for other cancers, the detection of marker genes alone is insufficient to distinguish neoplastic and nonneoplastic cells. For example, in a study of pancreatic ductal carcinoma, clustering analysis produced multiple cell clusters identified as ductal cells based on the expression of ductal marker genes^56^. Without additional information, this clustering analysis alone was unable to determine the malignant status of the identified ductal cell clusters. Although the upregulation of aberrant expression programs such as cancer-associated pathways (e.g., angiogenesis and proliferation) may implicate certain cell clusters, annotations based on pathway expression alone may be ambiguous. This is because neoplastic cells can also express genes and pathways typically associated with canonical nonneoplastic cells in ways that we might not expect. For example, an scRNA-seq analysis of glioblastoma identified transcriptionally distinct neoplastic subpopulations exhibiting the upregulation of transcriptional programs associated with expected oncogenic programs such as oncogenic signaling, proliferation, and hypoxia. However, the same analysis identified another subpopulation of neoplastic cells exhibiting the upregulation of complement/immune response programs typically associated with immune cells^57^. As such, orthogonal evidence beyond marker gene or pathway expression is often needed to confidently distinguish between neoplastic and nonneoplastic cells.

To this end, computational methods have been developed to identify DNA-level aberrations directly from scRNA-seq data (Fig. 4b). Large-scale copy-number variations (CNVs) can be inferred by comparing the smoothed averaged gene-expression profiles of neoplastic cells harboring CNVs to an appropriate normal tissue reference^57,58^. The presence of deletions or amplifications will on average lead to reduced or increased expression of genes, respectively, within affected loci compared to copy-neutral reference expression for the same cell type. Hierarchical clustering of smoothed normalized expression magnitude deviations can distinguish cells harboring CNVs from normal diploid cells. In a study of pancreatic ductal carcinoma, such expression-based CNV inference was used to show that one ductal cell cluster exhibited higher CNV levels than another ductal cell cluster; in combination with the upregulation of aberrant cancer-related programs such as cell proliferation, migration, and hypoxia, these findings implicated the former ductal cell cluster as the malignant subpopulation^56^. Overall, CNV inference from scRNA-seq data has been applied to distinguish neoplastic and nonneoplastic cells in many cancers, including a variety of gliomas^46,57,59–61^, melanoma^45^, head and neck cancer^47^, breast cancer^62^, and multiple myeloma^58^.

However, the reliability of such expression-based CNV inference is dependent on how well the cancer expression profile is matched to the normal reference, in terms of both technical and biological factors^58^. An appropriate normal reference is needed to ensure that the observed deviations in expression magnitude are the result of underlying copy number changes rather than platform- or cell-type-specific differences. The identification of an appropriate normal reference may be particularly challenging if the cancer cell type of origin is unknown. An alternative computational approach for identifying CNVs is based on the variant allele frequencies (VAFs) of heterozygous germline single-nucleotide polymorphisms (SNPs)^58^. Although most scRNA-seq studies focus on gene-expression counts, scRNA-seq also provides information about SNPs by virtue of sequencing-based data. Changes in copy number skew the observed VAFs^12^ in scRNA-seq data such that the presence of deletions leads to the persistent depletion of the lost allele, while amplification will lead to increased abundance of the amplified allele on average. Since allele-based approaches rely on high coverage of many SNP sites, data from scRNA-seq protocols that can achieve full-transcript coverage (e.g., Smart-seq2) are best for these analyses. In contrast, the analysis of data from high-throughput scRNA-seq protocols that capture only the 3′ or 5′ transcript ends will be limited to the identification of larger whole-chromosome and chromosome-arm-scale alterations. Furthermore, allele-based approaches will not be able to confidently distinguish copy-neutral loss-of-heterozygosity from deletions or to distinguish different numbers of copy-number amplifications. Integrating allelic and expression information can overcome these limitations and achieve more robust probabilistic CNV inference^58^.

Nevertheless, some cancers do not harbor such large-scale CNVs. Other smaller-scale DNA-level alterations such as somatic point mutations can also be identified from scRNA-seq data and used to distinguish neoplastic cells (Fig. 4c). However, the detection of somatic point mutations from scRNA-seq data is limited to mutations within expressed exons at sites with sufficient read coverage. This lack of coverage at the mutation site of interest is a particular limitation for scRNA-seq protocols involving 3′ or 5′ rather than full-transcript sequencing. Likewise, both technical and biological factors that result in the selective detection of a nonmutant allele (e.g., uneven amplification^63^, prevalent stochastic mono-allelic expression and detection^64,65^, and allelic exclusion^66^) limit our ability to confidently call heterozygous point mutations using scRNA-seq alone^46,67^. As such, alternative protocols and technologies have been developed to combine scRNA-seq with targeted locus-specific amplification^46,68,69^ or targeted quantitative polymerase chain reaction-based mutation detection^61,67^ to enable the robust detection of selected point mutation status directly from or in conjunction with scRNA-seq data. Furthermore, in the calling of somatic point mutations from scRNA-seq data, false positives that may be caused by RNA editing must also be considered. The reduction of false positives may be achieved by limiting the analysis to the mutations and variants identified through the WES of the same tumor sample or to mutations known to be recurrent in relevant cancers from databases such as COSMIC^70^. Some computational approaches have also been specifically designed to call point mutations from RNA-seq^71^ and scRNA-seq data^72,73^ while taking into consideration such potential false positives and negatives.

Beyond distinguishing neoplastic cells from nonneoplastic cells, CNV inference and somatic mutation calling can be used to distinguish genetically distinct neoplastic subclones. Notably, by inferring such alterations from scRNA-seq data, the transcriptional profiles of genetic subclones can be directly compared to characterize the transcriptional consequences of observed genetic alterations (Fig. 4d). Nevertheless, studies have shown that transcriptional heterogeneity among neoplastic cells does not necessarily reflect observed genetic relationships^58,67,74,75^, highlighting the need for the further assessment of the interplay of genetic and transcriptional heterogeneity.

Nevertheless, some cancers are not well defined by either large-scale CNVs or somatic point mutations. For example, chronic myeloid leukemia (CML) cells are generally defined by the presence of the *BCR-ABL* fusion gene. While gene fusions may be detected in data generated with full-transcript scRNA-seq protocols (e.g., SmartSeq2), limitations in detection sensitivity can result in false negatives^76^. To robustly detect gene fusions, the scRNA-seq library preparation protocol can be modified to include primers for the targeted amplification of specific gene fusions. One study of CML successfully applied this approach with a primer targeting the *BCR-ABL* fusion gene to confidently distinguish CML cells^76^. Ultimately, a combination of these approaches should be used and can even be integrated with machine-learning classifiers^69^ to identify *bona fide* neoplastic cells.

### Inferring communication with the tumor microenvironment

Neoplastic cells exist among the heterogeneous composition of nonneoplastic cell types and states within a tumor microenvironment that may contribute to tumor evasion and progression^77^, angiogenesis^78^, and therapeutic resistance^79^. scRNA-seq provides the opportunity to characterize the many cell types in the tumor microenvironment, from stromal fibroblasts to diverse immune subtypes, in a high-throughput and mostly unbiased manner. In a study of the lung tumor microenvironment, scRNA-seq analysis identified functionally distinct fibroblast subtypes as well as remodeled tumor endothelial cells that downregulate antigen presentation and contribute to immune tolerance and suppression^43^. Similarly, the scRNA-seq analysis of tumor-associated macrophages has been used to distinguish between ﻿proinflammatory macrophages that resist tumor progression and tissue-reparative macrophages that promote tumor growth and metastasis in breast cancer^80^, lung cancer^81^, and hepatocellular carcinoma^32^. Furthermore, the scRNA-seq analysis of tumor-infiltrating T cells has identified notable subpopulations in different cancers that may be potential targets for novel immune checkpoint inhibitors, including exhausted T cells in lung cancer^43^, tissue-resident memory T cells in breast cancer^82^ and melanoma^83^, and regulatory T cells in non-small-cell lung cancer^84^ and colorectal cancer^85^.

**Fig. 5:**
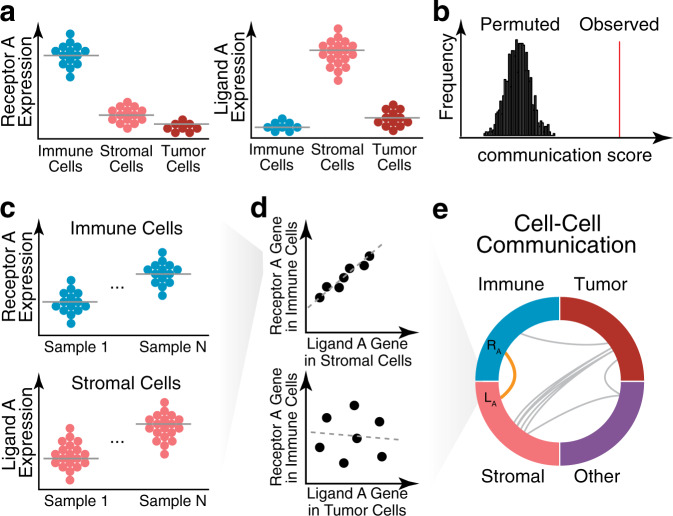
Inference of cell–cell communication. **a** The codetection of receptor-ligand pairs may be used to identify putative cell-cell communication. In this illustration, the single-cell expression levels of known receptor-ligand pairs (Receptor A and Ligand A) are shown across cell types. High receptor expression is identified in immune cells, as illustrated in the beeswarm plot, where each point is a cell. Likewise, high ligand expression is identified in stromal cells. Such codetection may indicate putative cell–cell communication between these two cell types. **b** Codetection may be quantified as a cell–cell communication score that is evaluated through permutation testing to assess statistical significance. **c** When multiple samples are available, correlation between receptor-ligand pairs may be used to identify putative cell–cell communication. In this illustration, the single-cell expression levels of known receptor–ligand pairs (Receptor A and Ligand A) are again plotted for N samples. **d** (top) The average Receptor A gene expression in an immune cell type versus the average Ligand A gene expression in a stromal cell type shows a high correlation across samples (represented as points), indicative of cell–cell communication between these two cell types. (bottom) In contrast, the correlation of the average Receptor A gene expression versus the average Ligand A gene expression in immune and tumor cells shows a poor correlation across samples. **e** Such correlations may be indicative of cell–cell communication between immune and stromal cell types (orange). Such testing can be applied to all cell-type pairs and visualized as a circle plot.

Beyond characterizing heterogeneity in the tumor microenvironment, computational methods have also been developed to infer putative communication between different cell types. Since scRNA-seq approaches require single-cell suspensions, the spatial context of the cell arrangement in the original tissue is lost. Therefore, computational methods for inferring cell-cell communication from scRNA-seq data require evidence without information on the spatial proximity of cells. To infer putative communication between cell types, cell–cell communication methods have generally relied on the comparison of the expression levels of a receptor gene in one cell type and a corresponding ligand gene in another cell type^47,86–88^ using a curated list of known receptors and corresponding ligands^89^ (Fig. 5a). For example, for each known receptor–ligand pair, CellPhoneDB calculates the mean expression of the receptor gene in one cell type and the mean expression of the ligand gene in another cell type^88^. These observed means are then assessed for statistical significance by comparing them to a null distribution, where means are recomputed after randomly permuting the cell-type labels of all cells (Fig. 5b). A graph-based approach for generating a null distribution has also been used to assess statistical significance^90^. When analyzing a large number of scRNA-seq datasets, putative communication can also be identified by computing the correlation of receptor gene expression in one cell type with the corresponding ligand gene expression in another cell type across all scRNA-seq datasets^90^ (Fig. 5c–e). More recently, these ideas have been extended by using a computational method known as NicheNet^91^, which integrates gene expression data with prior knowledge of intracellular signaling and gene regulatory networks. This method identifies ligands in one cell type associated with the expression of genes downstream of the corresponding receptor in another cell type.

Approaches focused on scRNA-seq datasets alone can be limited in terms of their statistical power due to the limited number of patients and samples profiled. To take advantage of the greater availability of large collections of bulk RNA-seq samples, computational deconvolution approaches have been developed to infer the proportions of different immune and stromal cell bulk RNA-seq samples after the identification of cell-type-specific markers from scRNA-seq data^92^. The fundamental assumption of deconvolution is that a bulk sample is a mixture of multiple transcriptionally distinguishable cell types. Most deconvolution methods model the bulk gene-expression matrix as the product of an scRNA-seq gene expression reference (observed) and estimated cell-type proportions for all samples (unobserved) using different types of regression models, such as linear regression^93^ or support vector regression^94,95^. Different approaches for cell-type marker gene selection approaches can influence the accuracy of cell-type proportion estimates. Marker genes can be selected in many ways, including differential expression analysis or the use of the Gini index^96^. Likewise, methods can weight genes by variability^97^ or incorporate ﻿patient-specific covariance^98^ to improve marker selection. Accurate proportion estimation for transcriptionally similar cell types is particularly challenging. To address the collinearity of gene expression profiles for similar cell types, one approach is to remove the union of the most highly expressed genes among similar cell types from marker selection^93^. Another approach is to first estimate the proportion of a group of transcriptionally similar cell types and then recursively select markers and obtain estimates for finer cell types within each group^98^. Such deconvolution approaches have been applied to estimate the proportion of infiltrating immune cells for 23 cancer types from The Cancer Genome Atlas (TCGA), in which increased immune infiltration was found to be associated with longer median survival^93^. An important caveat to consider in any deconvolution analysis is that cancer cells may aberrantly express genes associated with canonical immune or nonneoplastic cell types. Therefore, to achieve accurate proportion estimates, the incorporation of neoplastic cells in marker gene selection is necessary to help ensure that the detection of marker genes reflects the underlying proportions of immune cells rather than aberrant expression by neoplastic cells.

### Delineating tumoral and microenvironmental evolution

While single-cell transcriptomic profiling techniques such as scRNA-seq offer transcriptome-wide molecular measurements at a single-cell resolution, these measurements ultimately represent a single snapshot in time. This lack of temporal information is particularly limiting for the study of cancer and other dynamic processes due to the continuous nature of cancer evolution and, more broadly, cellular development. Although scRNA-seq provides a snapshot of each individual cell at a single point in time, a snapshot of many cells representing a range of evolutionary stages can allow us to order these cells in pseudotime and within trajectories. To infer this pseudotime ordering of cells within putative trajectories, a number of computational trajectory inference methods have been developed^99–101^, compared^102^, and reviewed^74,103^. In the context of cancer, trajectory inference analysis has been applied to scRNA-seq data from healthy and cancerous kidneys. The analysis identified two divergent transcriptional trajectories, one of which corresponds to the development of nephrogenic rest cells and the other to Wilms cancer cells originating from cells of the ureteric bud, consistent with the hypothesis that Wilms tumor cells develop from aberrations in fetal nephrogenesis^104^. Likewise, trajectory inference analyses of infiltrating T cells in liver cancer^32,105^ and small-cell lung cancer^84^ have identified cellular state transitions between proliferating/activated and exhausted states.

**Fig. 6:**
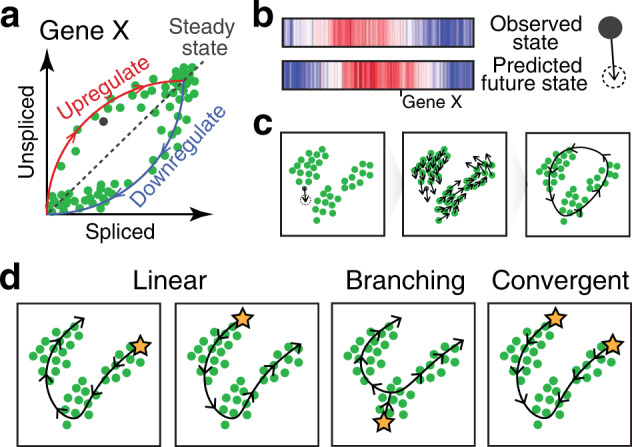
Trajectory inference combined with RNA velocity analysis. **a** Transcriptional dynamics model used to estimate RNA velocity. For an individual gene such as Gene X, RNA velocity is modeled across a population of cells based on the observed spliced (e.g., exonic) and unspliced (e.g., intronic) mRNA abundances, plotted on the *x*- and *y*-axes, respectively. In this model, cells, illustrated as points, upregulating expression of the gene are expected to exhibit a relatively higher proportion of unspliced mRNA compared to spliced mRNA, while cells downregulating expression of the gene are expected to exhibit a relatively lower proportion of unspliced mRNA compared to spliced mRNA. **b** Such models of transcriptional dynamics can be used to extrapolate expression levels for many genes at a future time point. In this illustration, the current high-dimensional observed transcriptional state for a cell is visualized in a heatmap in which each column represents a gene, where red indicates higher expression, and blue indicates lower expression. The predicted future transcriptional state for the same set of genes based on the RNA velocity model is shown below. **c** The future transcriptional state for each cell, as predicted by the RNA velocity models, can be projected to a lower-dimensional embedding (e.g., tSNE and PCA). An arrow can be used to connect the observed transcriptional state and the future predicted transcriptional state in the lower-dimensional embedding to visualize velocities. This can be performed for each cell individually, as a gridded velocity field, or as a single directed principal curve, as illustrated. **d** RNA velocity analysis may be applied to distinguish between different trajectory patterns, such as a linear progression through different states, versus branching or convergent trajectories, as illustrated. RNA velocity analysis may also be applied to identify the roots or origins of cellular trajectories, illustrated here as stars.

While trajectory inference methods are able to position cells along some axes, current methods do not estimate the underlying temporal kinetics regarding the rate or direction of progression through inferred trajectories. Prior knowledge regarding gene expression patterns may be useful for establishing the directionality of trajectories representing normal developmental processes, where we can assume that a trajectory starts from cells expressing stemness-related pathways and ends at cells expressing maturation-related pathways. However, such assumptions may no longer be valid in a cancer setting. RNA velocity analysis can address these limitations by providing directionality to inferred trajectories. RNA velocity analysis utilizes the relative ratio between intronic (i.e., unspliced, immature) and exonic (i.e., spliced and mature) reads in scRNA-seq data to infer the rate of change in transcript abundance to estimate the future transcriptional state of a cell^106^ (Fig. 6a, b). The observed versus the predicted future transcriptional state for each cell or group of transcriptionally similar cells can be projected onto inferred trajectories to provide putative directionality, which may be particularly useful in rooting trajectories as well as distinguishing between divergent versus convergent evolutionary trajectories (Fig. 6c, d). For example, the application of RNA velocity analysis to dendritic cells (DCs) from hepatocellular carcinoma suggested that two different conventional DC subpopulations have the potential to converge and transition into *LAMP3*+ tumor-associated DCs^32^.

While the application of trajectory inference and RNA velocity analysis offers the potential to identify the altered mechanisms of cell development in cancer pathogenesis, a number of precautions should be considered when applying such analyses in a cancer setting, especially when interpreting results for neoplastic cells. Trajectory inference relies on the adequate representation of cells in different developmental stages, such that the absence of intermediate stages may distort the inferred temporal dynamics^107^. This problem may be particularly pronounced in cancer, where scRNA-seq may capture multiple transcriptionally distinct subpopulations of cells but not the ancestral cells that gave rise to these populations^74^. Despite these challenges, the application of RNA velocity analysis to isocitrate dehydrogenase (IDH) wild-type glioblastoma cells has putatively identified an intermediate glioma stem-like cell (GSC) subpopulation that may transition from a mesenchymal to a proneural phenotype, implicating mesenchymal GSCs as the progenitors of proneural GSCs in IDH wild-type glioblastomas^108^. Nevertheless, RNA velocity analysis assumes that increased relative intronic expression reflects the presence of unspliced nascent transcripts. In a cancer setting, however, mutations in the splicing machinery may cause aberrant alternative splicing, resulting in differentially regulated intronic retention that violates this assumption. For example, in chronic lymphocytic leukemia as well as other myeloid neoplasms, recurrent mutations in splicing factor genes such as *SF3B1*, *U2AF1*, *SRSF2*, and *ZRSR2* have been observed^109^ and shown to cause a wide variety of aberrant alternative splice variants^110,111^. In such a scenario, RNA velocity models should explicitly avoid introns known to be impacted by aberrant splicing by excluding these introns from unspliced gene quantification or removing these genes from the model altogether.

## Discussion and outlook

The application of single-cell transcriptomics in cancer presents a number of unique analytical challenges and opportunities. In this review, we focused on emerging themes in the computational analysis of single-cell transcriptomics data in cancer research, highlighting unique challenges and opportunities.

However, despite the promise of single-cell transcriptomics and opportunities for computational method development in cancer research, discoveries will always be fundamentally limited by what can be measured. Notably, although a variety of protocols exist for scRNA-seq analysis, nearly all involve poly-A selection, thereby limiting the ability to examine non-polyadenylated transcripts, such as small nucleolar RNAs, histone mRNAs, pre-mRNAs, and long noncoding RNAs, which may play diverse regulatory roles in cancer^3^. Even within poly-A selection scRNA-seq protocols, droplet-based protocols that are restricted to only 3′ or 5′ ends will inherently be more limiting for allele-based CNV inference, mutation calling, and fusion gene detection compared to full-transcript single-cell RNA-seq protocols. Furthermore, some cell types (e.g., neutrophils, epithelial cells, and neurons) may not be compatible with the dissociation, encapsulation, or other processing steps of all scRNA-seq protocols. Alternative protocols such as single-nucleus RNA-seq^112^ (snRNA-seq) may be applied to these cell types and to cells from frozen specimens. Different sample preservation techniques, such as the freezing or formalin fixed-paraffin embedding of tissues commonly used for cancer samples, may require different protocols and introduce different limitations^113^. Future unified analyses of the same cancer samples with protocols (whole cell vs. nuclei, fresh vs. frozen) will help to further elucidate the precise limitations and biases introduced by each protocol. As always, care must be exercised in selecting the protocol that is best able to address the question of interest while remaining aware of its inherent limitations, to avoid drawing spurious biological conclusions.

Although transcriptional heterogeneity has been observed in a variety of cancers, the extent to which this transcriptional heterogeneity can be mapped to underlying genetic, epigenetic, or spatial contextual causes and their interplay remains unclear. While the transcriptional impact of genetic variants may be assessed to some degree through the direct inference of genetic information from scRNA-seq data, as previously discussed, the identification of associations with other aspects of heterogeneity such as epigenetic or spatial heterogeneity may require the integration of additional data, technologies, and computational methods. For example, to investigate the role of epigenetic heterogeneity and its interplay with transcriptional heterogeneity at the single-cell level, a number of multiomic computational analysis approaches^29,114^ have been developed to enable unified analysis across transcriptomic and epigenetic data modalities, albeit for different cells. However, these approaches generally rely on linking functions to perform mapping between data modalities, such as mapping between gene expression and promoter or gene body accessibility under the assumption that greater accessibility correlates with a higher gene expression magnitude. In a cancer setting, however, these assumptions may no longer be valid when regulatory factors are mutated. As such, greater consideration may be necessary to ensure that linking functions are appropriate when applying such computational data integration approaches in a cancer setting. New technologies and protocols are also being developed to enable multimodal measurements, including simultaneous transcriptomic and epigenomic measurements, within a single cell^53,115,116^. In conjunction with novel computational analysis approaches, such technologies offer the potential to contribute to our growing understanding of these different aspects of cancer heterogeneity and their interplay. Likewise, to investigate the role of the spatial context, a number of imaging a number of imaging technologies have been developed technologies have been developed to enable the targeted spatially resolved single-cell transcriptomic profiling of 100–1000 s of genes^117–119^. More recently, these technologies have been expanded to a near-genome-wide scale^120–122^. We anticipate that such spatially resolved single-cell transcriptomic data generated from these different technologies will require new computational pipelines and methods for proper processing (e.g., RNA decoding and cell segmentation), quality control, and normalization. Furthermore, the computational methods for analyzing scRNA-seq data may need to be modified to develop in situ analogs appropriate for spatially resolved single-cell transcriptomics data. RNA velocity analysis of scRNA-seq data that leverages the ratio of intronic and exonic gene expression has been modified to create an in situ analog that leverages the ratio of nuclear and cytoplasmic gene expression^120^. In conjunction with novel computational analysis approaches, these emerging technologies offer the potential to contribute to our growing understanding of these different aspects of cancer heterogeneity and their interplay.

As the numbers of analyzed cells and samples continue to increase exponentially, particularly because of international and collaborative efforts such as the Human Cell Atlas^123^, Human Developmental Cell Atlas, Pediatric Cell Atlas^124^, HuBMAP^125^, Human Tumor Atlas Network, LifeTime EU Flagship, and others, there is a need to improve the scalability of computational methods through implementation improvement and algorithmic optimization. While the application of these computational methods to a cancer setting may present a number of unique challenges, additional efforts are ultimately needed to iterate between data-driven hypothesis generation and the orthogonal validation of computational predictions. Despite these challenges, single-cell transcriptomics analysis presents tremendous opportunities to contribute to our understanding of cancer heterogeneity, pathogenesis, evolution, and microenvironmental interactions to lay a foundation for new therapeutic innovations.
